# Integrated Network Pharmacology and UPLC/Q-TOF-MS Screen System to Exploring Anti-Inflammatory Active Components and Mechanism of Shunaoxin Pills

**DOI:** 10.1155/2022/2868767

**Published:** 2022-04-14

**Authors:** Nianwei Chang, Yu Wang, Min Jiang, Gang Bai

**Affiliations:** ^1^Tianjin University of Traditional Chinese Medicine, Tianjin, China; ^2^State Key Laboratory of Medicinal Chemical Biology, College of Pharmacy and Tianjin Key Laboratory of Molecular Drug Research, Nankai University, Tianjin, China

## Abstract

**Background:**

Chronic cerebral ischemia (CCI) is a pathological condition associated with a variety of cerebrovascular diseases. Shunaoxin pills (SNX) are a traditional Chinese medicine (TCM) used to improve blood circulation. However, its multicomponent and multitarget features make it difficult to decipher the molecular mechanisms.

**Objective:**

Thus, in this study, we aimed to identify the key anti-inflammatory components of SNX as markers for standardization and quality control and the potential pharmacological mechanisms of SNX in the treatment of CCI by network pharmacology to provide scientific evidence of its clinical efficacy.

**Methods:**

We evaluated the anti-inflammatory effect of SNX using ultra-high performance liquid chromatography-quadrupole time-of-flight mass spectroscopy (UPLC/Q-TOF-MS) combined with a dual-luciferase reporter assay for nuclear factor kappa B (NF-*κ*B) inhibition to identify the active components in SNX. In addition, key pathways involved in the anti-inflammatory effect of SNX were predicted using a network pharmacology approach, and some crucial proteins and pathways were further validated by Western blotting.

**Results:**

Shunaoxin pills inhibited NF-*κ*B through tumor necrosis factor-*α* (TNF-*α*) stimulation in 293T cells. The therapeutic effect may be related to 10 pathways regulated by ligustilide, ferulic acid, ligustrazine, and senkyunolide I. It was further confirmed that ligustilide could reduce the inflammatory response by inhibiting the phosphorylation of p38 and 3-phosphoinositide-dependent kinase 1 (PDK1).

**Conclusions:**

Ligustilide, senkyunolide I, ferulic acid, and ligustrazine could be used as anti-inflammatory Q-markers to control the quality of SNX, and p38 and PDK1 might be potential targets of SNX in the treatment of CCI.

## 1. Introduction

Traditional Chinese medicine (TCM) has been used to maintain health and treat diseases for thousands of years in China, and its use has been increasing globally in the recent decades [1]. Moreover, the World Health Organization has recognized TCM as an influential global medical compendium, which means that TCM has been widely accepted worldwide [2]. The largest application category of TCM is Chinese herbal medicine (CHM), which comprises sliced herbal and Chinese patented drugs. Traditional Chinese medicine preparations are generally composed of various TCMs. A large number of patients have used TCM as an alternative therapy because of its effectiveness and lack of serious side effects. However, TCM still lacks competitiveness in the international market, mainly due to the lack of knowledge on the active components and mechanisms of TCM, making it difficult to develop a scientific and effective evaluation system for the efficacy and safety of TCM. Modern research has shown that quality analysis based on analytical methods such as quantitative analysis, chromatographic fingerprinting, and identification analysis cannot effectively reflect the consistency of the quality and curative effects of TCM [3, 4]. Therefore, linking biological activity with the chemical composition of TCM is currently the most important challenge for quality control.

Chronic cerebral ischemia is a long-term cerebral insufficiency caused by a vascular disease that may evolve into senile vascular dementia or Alzheimer's disease [5]. The inflammatory process is considered to be the most important cause of the many etiologies that thought to be responsible for the development of CCI. The inflammatory process exists in all steps of the ischemic cascade and even participates in stroke-induced brain damage [6]. Nonsteroidal anti-inflammatory drugs and antibiotics are usually used to induce inflammatory responses in ischemic tissues due to the excessive activation of neutrophils. However, these drugs may cause systemic immune suppression, leading to severe side effects and susceptibility to infections [7–9]. Shunaoxin pills (SNX) have been widely used to treat cerebrovascular diseases in China, including *Ligusticum chuanxiong* Hort. and *Angelica sinensis* (Oliv.) Diels, both of which improve chronic cerebral ischemia (CCI) [10, 11]. Analytical studies have reported that the primary components of SNX include lactones (such as 4-OH-3-butylphthalide, senkyunolide I, and ligustilide), phenolic acids (such as ferulic acid and caffeic acid), and alkaloids (such as ligustrazine) [12], and most of them have antioxidant and anti-inflammatory effects [13–16]. However, as a compound preparation, whether the main active ingredients are changed due to the preparation process and how to exert the anti-inflammatory mechanism are problems that need to be urgently addressed.

In this study, we aimed to screen the anti-inflammatory active components of SNX using ultra-high performance liquid chromatography-quadrupole time-of-flight mass spectroscopy (UPLC/Q-TOF-MS) and dual-luciferase reporter assays and to determine their targets and pathways in the treatment of CCI via network pharmacology methods. This study aims to propose a research strategy to combine the active ingredient screening system with network pharmacology to explore the Q-markers with different activities in TCM.

## 2. Materials and Methods

### 2.1. Materials

Shunaoxin pills (100 mg, the amount ratio of *Ligusticum chuanxiong* Hort. and *Angelica sinensis* (Oliv.) Diels was 1 : 1; Tianjin Zhongxin Pharmaceutical Group Co., Ltd., Tianjin, China, batch number 677014) were dissolved in 10 mL of methanol and used for UPLC analysis. Ferulic acid, ligustilide, ligustrazine, and senkyunolide I (Tianjin Yifang S&T Co., Ltd., Tianjin, China) with a purity ≥98%, as determined by high performance liquid chromatography (HPLC), and dexamethasone (Dex) (Bayer Vital GmbH, Leverkusen, Germany) were dissolved in Dulbecco's modified Eagle medium (DMEM; Gibco, Grand Island, NY, USA) with 0.1% dimethyl sulfoxide (DMSO; Solarbio, Beijing, China) and used for the anti-inflammatory activity studies.

### 2.2. Cell Culture

Mouse brain microvascular endothelial cells (bEnd3) and human embryonic kidney 293 T cells (HEK 293) were obtained from the American Type Culture Collection (Manassas, VA, USA). Both cells were cultured in high-glucose DMEM with 10% fetal bovine serum (FBS, Sigma Corporation, MO, USA) and 1% penicillin-streptomycin (100x, Gibco BRL Life Technologies, NY, USA) solution at 37°C and placed in a 5% carbon dioxide (CO_2_) thermostatic incubator.

### 2.3. Dual-Luciferase System for Nuclear Factor Kappa B (NF-*κ*B) Inhibition Assay

The dual reporter gene assay for studying the NF-*κ*B transcription factor was performed as described in the literature [17]. The cell experiments were divided into six groups (*n* = 6): the control group, model group, Dex group (10^−6^ mol·L^−1^), and three SNX groups with different concentrations (10^−2^, 10^−3^, and 10^−4^ g·mL^−1^). Dexamethasone served as a positive control drug in the experiment. The five groups (except the control group) were simultaneously stimulated with TNF-*α* (10 ng·mL^−1^) for 6 h and then administered with DMEM, different doses of SNX, or Dex for 6 h. The cells were then washed with phosphate buffered saline (PBS), lysed, and tested by dual-luciferase reporter assays according to the manufacturer's instructions.

### 2.4. UPLC/Q-TOF-MS Analysis

The sample was analyzed using a UPLC/Q-TOF-MS system (Waters MS Technologies, Manchester, UK), and the settings of the instrument parameters were as described by Chang et al. [12]. The injection volume was 2.0 *μ*L with a gradient elution of acetonitrile (A) and 0.1% formic acid aqueous solution (B) using the following protocol: 2–27% A from 0 to 10 min, 27–33% A from 10 to 18 min, 33–33.5% A from 18 to 20 min, 33.5–38% A from 20 to 22 min, 38–54% A from 22 to 24 min, 54–69% A from 24 to 26 min, 69–71% A from 26 to 28 min, 71–73% A from 28 to 30 min, 73–100% A from 30 to 33 min, and 100–2% A from 33 to 35 min. The flow rate was 0.40 mL/min.

### 2.5. The Screening and Verification of Active Components in SNX

Fractions of SNX between the UPLC and MS were gathered into a 96-well plate every 1 min and then evaporated until dry using a vacuum drying oven. Then, 0.1 mL of cell culture medium was added to each well, and ultrasonic vibration was applied for 10 min. The cell culture medium containing the fractions was stored at –20°C for the screening of the active ingredients.

To verify the accuracy of the screened components, the effects of the active ingredients of ferulic acid, ligustilide, ligustrazine, and senkyunolide I (10^−4^–10^−6^ mol·L^−1^) were determined.

### 2.6. Prediction of Targets and Pathways of Anti-Inflammatory Components in SNX

The three-dimensional structures of the effective active components in SNX were entered into the PharmMapper database (http://59.78.96.61/pharmmapper) to predict the targets. The target genes for CCI in this study were collected from the Gene Cards (https://www.genecards.org/) and Online Mendelian Inheritance in Man (OMIM) (http://www.omim.org/) databases. Protein–protein interactions (PPIs) were analyzed using the String 11.0 database (http://www.string-db.org/). The pathways were determined via Kyoto Encyclopedia of Genes and Genomes (KEGG) pathway analysis (https://www.kegg.jp).

### 2.7. Verification of the Signaling Pathway of Ligustilide

The bEnd3 cells were grown in six-well plates for 24 h and stimulated with 20 ng mL^−1^ of TNF-*α* for 6 h. The cells were then treated with ligustilide (10^−5^–10^−7^ mol·L^−1^) for 6 h. The cells were lysed in radio immunoprecipitation assay buffer for 30 min on ice. The protein concentration was determined using the bicinchoninic acid protein assay kit (Solarbio, Beijing, China). The proteins were separated by 12% sodium dodecyl sulfate polyacrylamide gel electrophoresis (SDS-PAGE) and transferred to polyvinylidene fluoride (PVDF). The membranes were blocked with 5% nonfat dry milk at 24–26°C for 1 h and then incubated with primary antibodies against pyruvate dehydrogenase kinase 1 (PDK1), phosphorylated (p)-PDK1, p38, and p-p38 (CST, MA, USA) overnight at 4°C. The membranes were washed with Tris-buffered saline with 0.1% Tween 20 detergent (TBST) for 30 min, incubated with a secondary antibody (CST, MA, USA) at 24–26°C for 3 h, and washed again. The membranes were incubated with chemiluminescent horseradish peroxidase substrates and exposed to ECL plus Western blotting detection reagents (GE Healthcare, Buckinghamshire, UK).

### 2.8. Statistical Analysis

All data were expressed as mean ± standard deviation (SD). The *t*-tests were used for statistical comparisons between the groups. All statistical analyses were performed using Bonferroni corrections and a statistical analysis software (GraphPad Prism 7.0). Statistical significance was set at *P* < 0.05.

## 3. Results

### 3.1. Effects of SNX on NF-*κ*B Inhibition

**Figure 1:**
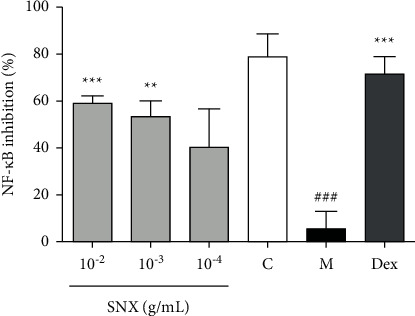
Effects of SNX on NF-*κ*B inhibition. Effects of the three doses of SNX on the level of NF-*κ*B in TNF-*α*-stimulated HEK 293 cells. The results are expressed as mean ± SD. Compared with the model group, ^*∗∗∗*^*P* < 0.001 and ^*∗∗*^*P* < 0.01, and compared with the control group, ^###^*P* < 0.001. Isolation and identification of active ingredients in SNX are performed using bioactivity-based UPLC/Q-TOF-MS analysis.

In order to clarify the active substances, SNX extracts inhibiting NF-*κ*B activity were first verified. The effect of different doses of SNX groups on NF-*κ*B levels in HEK 293 cells is shown in Figure 1. Dex was used as a positive control for NF-*κ*B level detection. The high dose of SNX significantly inhibited NF-*κ*B production, which is induced by TNF-*α* (SNX, 10^−2^ g·mL^−1^, ^*∗∗∗*^*P* < 0.001; SNX, 10^−3^ g·mL^−1^, ^*∗∗*^*P* < 0.01). Therefore, SNX contains anti-inflammatory agents, which have a role at the cellular level that is consistent with its application in TCM.

**Figure 2:**
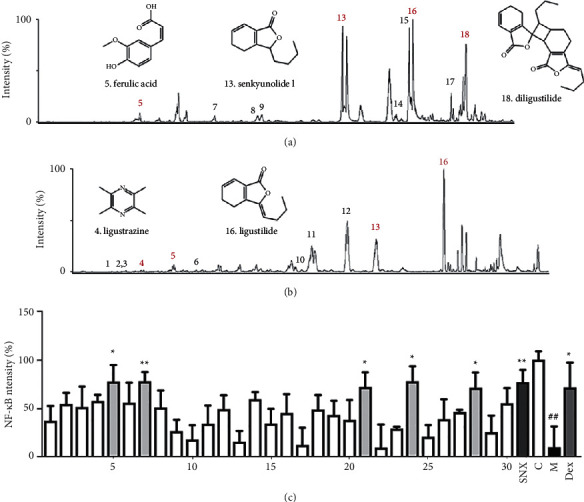
UPLC/Q-TOF-MS coupled with a dual-luciferase reporter assay utilized for the identification of active ingredients in SNX. (a) Total ion current (TIC) chromatograms in the positive ESI mode. (b) TIC chromatograms in the negative ESI mode. (c) The bioactivity chromatograms obtained via the luciferase reporter assay system for NF-*κ*B inhibition. The results are expressed as the mean ± SD. Compared with the model group, ^*∗*^*P* < 0.01 and ^*∗*^*P* < 0.05, and compared with the control group, ^##^*P* < 0.01.

SNX was utilized to isolate, screen, and identify NF-*κ*B inhibitors via UPLC/Q-TOF-MS with a luciferase reporter assay system. Figures 2(a) and 2(b) show the total ion current chromatograms in the positive and negative electrospray ionization (ESI) modes, respectively. Thirty fractions were tested for NF-*κ*B inhibition (Figure 2(c)).  Table 1 provides the detailed results of the identified bioactive compounds and MS/MS information. Five fractions showed probable NF-*κ*B inhibition (peaks 1–5). Among the five anti-inflammatory active substances, dimeric ligustilide, which was predicted by our previous studies, was found, but it might not be absorbed by the human body; therefore, it was not studied further.

**Figure 3:**
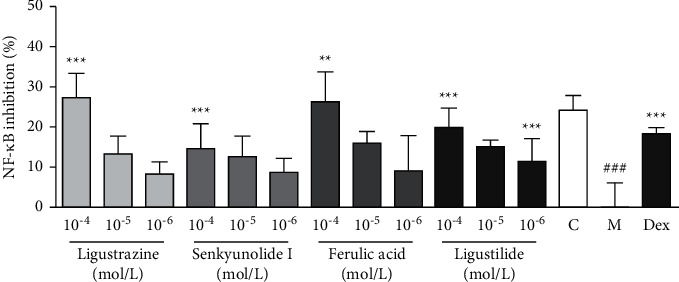
Confirmation of the effects of potential active ingredients in SNX. The results are expressed as the mean ± SD. Compared with the model group, ^*∗∗∗*^*P* < 0.001, ^*∗∗*^*P* < 0.01, and ^*∗*^*P* < 0.05, and compared with the control group, ^###^*P* < 0.001. The targets and pathway predictions of the active components of SNX.

To confirm the bioactivity of the compounds, various concentrations (10^−4^–10^−6^ mol·L^−1^) of four ingredients (senkyunolide I, ferulic acid, ligustilide, and ligustrazine) were selected and verified using the above method (Figure 3). The luciferase reporter assay result showed that senkyunolide I, ferulic acid, ligustilide, and ligustrazine inhibited the overexpression of NF-*κ*B in HEK 293 cells in a dose-dependent manner.

**Figure 4:**
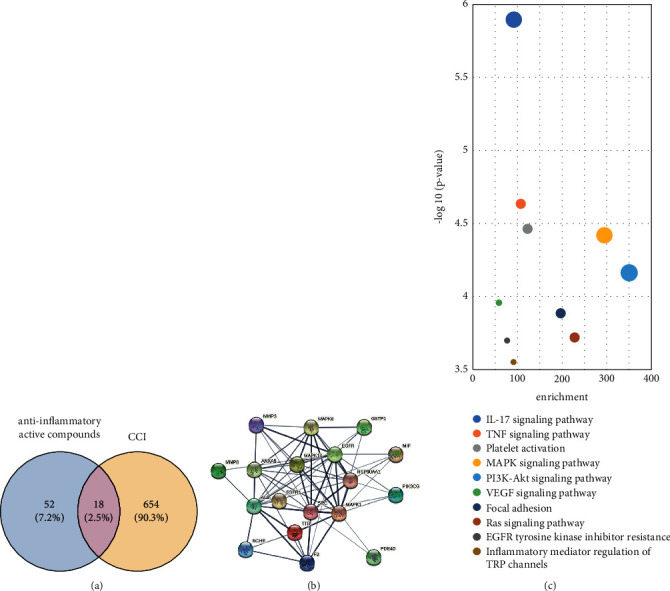
Network pharmacology analysis of the active components of SNX. (a) The 18 overlapping targets between the CCI and anti-inflammatory active compounds of SNX. (b) The PPI network of 18 overlapping targets. (c) The KEGG pathway enrichment analysis of 18 overlapping targets and the top 10 significantly enriched inflammation-related pathways.

To study the relationship between the bioactive compounds and the pathways and targets, we used PharmMapper and KEGG databases for virtual calculation. Ultimately, the data of 52 targets of four ingredients (senkyunolide I, ferulic acid, ligustilide, and ligustrazine) with absorption properties and 654 known therapeutic targets for CCI were obtained. By taking the intersection of the drug targets and disease targets, as shown in Figure 4(a), a total of 18 targets were predicted, which indicate that these genes could play a major role in the SNX of CCI. To clarify the potential mechanism of the anti-inflammatory effect of SNX on CCI, we constructed a PPI network (Figure 4(b)). To further explore whether the anti-inflammatory effects of SNX affect CCI through 18 targets, we performed a KEGG enrichment analysis. As shown in Figure 4(c), the KEGG pathway analysis of 18 targets revealed several inflammatory pathways correlated with the development and treatment of CCI, including the interleukin (IL)-17 signaling pathway, TNF signaling pathway, platelet activation, mitogen-activated protein kinase (MAPK) signaling pathway, phosphatidylinositol 3-kinase (PI3K)-protein kinase B (Akt) signaling pathway, vascular endothelial growth factor signaling pathway, focal adhesion, Ras signaling pathway, epidermal growth factor receptor tyrosine kinase inhibitor resistance, and inflammatory mediator regulation of transient receptor potential channels. The results show that the SNX pathway includes multiple targets, and the targets are also involved in multiple pathways. This complex regulatory relationship indicates that the anti-inflammatory active pharmacological components of SNX may be used to treat CCI through these signaling pathways.

**Figure 5:**
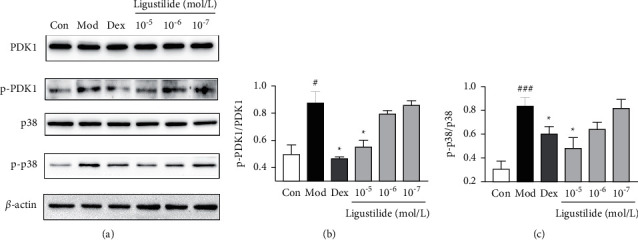
(a) Ligustilide attenuated TNF-*α*-induced phosphorylation of p38 and PDK1. (b) The relative intensity data of p-PDK1 to PDK1. (c) The relative intensity data of p-p38 to p38. The results are expressed as the mean ± SD. Compared with the model group, ^*∗*^*P* < 0.05, and compared with the control group, ^###^*P* < 0.001 and ^#^*P* < 0.05.

Ligustilide interfering with the expression of MAPK and PI3K-Akt pathways through network pharmacology predictions; ligustilide may regulate p38 (MAPK14) and PDK1 (PDPK1). The results are shown in Figure 5, and the phosphorylation of p38 and PDK1 increased in bEnd3 cells stimulated by TNF-*α*, whereas ligustilide reduced this high expression.

## 4. Discussion

Recombinant tissue plasminogen activator remains the first choice for the treatment of CCI [18, 19]. However, the treatment process may lead to ischemia-reperfusion injury, including destruction of the blood-brain barrier, brain edema, and hemorrhage [20]. The mechanisms of CCI injury are complex and include excitatory amino acid toxicity, calcium overload, inflammatory reactions, oxidative damage, and apoptosis [21–23]. Among the abovementioned mechanisms, an increasing amount of evidence shows that inflammation is an important pathogenic factor in CCI. In the early phase of cerebral ischemia, brain tissue that produces oxidative stress promotes the activation of NF-*κ*B, which regulates the transcriptional induction of various proinflammatory genes [24, 25]. Modern research shows that NF-*κ*B is activated, leading to the excessive production of proinflammatory cytokines, including TNF-*α*, IL-1*β*, and IL-6, and inducible nitric oxide synthase, which cause inflammatory injury on the basis of cerebral ischemia [26, 27]. However, in transient and permanent cerebral ischemia models, specific or nonspecific NF-*κ*B inhibitors could significantly reduce the area of cerebral infarction and improve the neurological deficits [28, 29].This was further supported by our observations in the current study, in which we found that SNX significantly inhibited the level of NF-*κ*B to decrease inflammation. Previous studies on SNX efficacy mainly focused on the cerebral vasodilation and antioxidants. This study first discussed the possible mechanism from the perspective of anti-inflammatory effects, which means weakening NF-*κ*B activation might represent a viable therapeutic strategy for CCI. The results further showed that senkyunolide I, ferulic acid, ligustilide, and ligustrazine had anti-inflammatory activities, which also suggest that SNX can regulate NF-*κ*B through multiple components to exert an anti-inflammatory effect.

In recent years, the studies have found that combined drugs acting on multiple targets simultaneously are more effective in controlling complex cerebral ischemia systems than drugs acting on individual molecular targets [30]. In this study, network pharmacology was used to analyze SNX, and the results revealed that three different structural types of anti-inflammatory active ingredients in SNX may act on different inflammation-related signaling pathways by regulating 18 targets to play an anti-inflammatory role in the treatment of CCI. An analysis of the target PPI network revealed that some protein targets were regulated by two or more components, which are the main potential anti-inflammatory targets in the interaction network. Among them, MAPK14 (p38) is an effective target for the treatment of CCI. Rosuvastatin can inhibit the activation of p38 to prevent damage to the blood-brain barrier during CCI [31]. The p38 inhibitors SB203580 and SB239063 could prevent neuronal death caused by excitatory amino acid poisoning and hypoxia [32]. Some natural products such as ginsenoside Rg1 and andrographolide also target p38 for the treatment of CCI [33, 34]. Through the prediction of network pharmacology, the main anti-inflammatory active ingredients in SNX could also regulate p38 and play a therapeutic role, which was confirmed by ligustilide.

Chronic cerebral ischemia is a multifaceted pathological process with a series of mechanisms; thus, it has many target signal proteins such as protein kinase C, processed sterol regulatory element-binding protein 1 [32, 35], and so on. In this study, the important transduction protein PDK1 in the PI3K/Akt signaling pathway was detected by Western blotting. PDK1 is an important protein in the PI3K/Akt signal transduction pathway. As a critical player in platelet-triggered ischemic stroke, PDK1 can increase intracellular Ca^2+^ in platelets, platelet activation, and aggregability after stimulation with collagen [36]. The neuroprotective effect of melatonin is mediated by Akt phosphorylation at the Thr308 site of the activation loop via PDK1 [37]. This also shows that PDK1, as a kinase, is currently one of the most attractive targets for drug development. The results showed that ligustilide could also play a pharmacodynamic role by regulating the phosphorylation of PDK1. This shows that there are more unknown target proteins that require further exploration.

In TCM preparations, a reasonable Q-marker should strictly focus on components that are closely related to safety and efficacy. To date, for most TCM, the quality is evaluated using one compound as a marker in the Chinese Pharmacopeia. In this study, an activity-oriented SNX quality control method was established, wherein senkyunolide I, ferulic acid, ligustilide, and ligustrazine could be used as potential anti-inflammatory Q-markers to control the quality of SNX. They not only belong to different chemical structures but also have potent anti-inflammatory activity. This study only screened the Q-marker for the anti-inflammatory effect of SNX, and other pharmacological effects may have different Q-markers, which need to be further studied. These findings provide a basis for the study of other pharmacological effects and mechanisms of SNX and provide a reference for drug development and system improvement.

## 5. Conclusions

In this study, by establishing a platform based on anti-inflammatory activity to screen the Q-markers of SNX, we found that senkyunolide I, ferulic acid, ligustrazine, and ligustrazine could be used as anti-inflammatory Q-markers for SNX quality control, and PDK1 and p38 could be the potential targets for SNX in the treatment of CCI, paving the way for the theory of the combination of quality control and activity of TCM.

## Figures and Tables

**Table 1:** MS data in (±) ESI modes and the identification results for SNX.

No.	tR (min)	Identification	Mode	m/z	MS/MS	Composition	Source	Absorbable capacity
1	2.49	Protocatechuic acid	Neg	153.0182	153 [M-H]^−^; 109 [M-H-COOH]^−^	C_7_H_6_O_4_	*Angelica sinensis* (Oliv.) Diels	√
2	3.05	Caffeic acid	Neg	179.0344	179 [M-H]^−^; 135 [M-H-COOH]^−^	C_9_H_8_O_4_	*Angelica sinensis* (Oliv.) Diels	√
3	3.17	Vanillic acid	Neg	167.0337	167 [M-H]^−^; 149 [M-H-H_2_O]^−^; 123 [M-H-COOH]^−^	C_8_H_8_O_4_	*Ligusticum chuanxiong* Hort.	√
4	5.26	Ligustrazine	Neg	135.0455	135 [M-H]^−^	C_8_H_12_N_2_	*Angelica sinensis* (Oliv.) Diels	√
5	6.42	Ferulic acid	Neg	193.0498	193 [M-H]^−^; 178 [M-H-CH_3_]^−^	C_10_H_10_O_4_	Both	√
6	8.26	Guaiacol	Neg	123.0459	123 [M-H]^−^	C_7_H_8_O_2_	*Ligusticum chuanxiong* Hort.	√
7	11.42	Bergapten	Pos	217.0501	217 [M+H]^+^; 189 [M+H-CO]^+^	C_12_H_8_O_4_	*Ligusticum chuanxiong* Hort.	√
8	13.87	Anisic acid	Pos	153.0541	153 [M+H]^+^; 137 [M+H-CH_3_]^+^	C_8_H_8_O_3_	*Ligusticum chuanxiong* Hort.	√
9	14.10	4-OH-3-Butylphthalide	Pos	207.1044	207 [M+H]^+^; 189 [M+H-H_2_O]^+^; 177 [M+H-C_2_H_5_]^+^	C_12_H_14_O_3_	*Angelica sinensis* (Oliv.) Diels	√
10	14.57	Isoeugenol	Neg	163.1138	163 [M-H]^−^	C_10_H_12_O_2_	*Ligusticum chuanxiong* Hort.	√
11	15.35	Chrysophanol	Neg	253.0714	253 [M-H]^−^; 219 [M-H-2OH]^−^; 205 [M-H-2OH-CH_3_]^−^	C_15_H_10_O_4_	*Angelica sinensis* (Oliv.) Diels	√
12	17.89	Spathulenol	Neg	219.0649	219 [M-H]^−^	C_15_H_24_O	*Angelica sinensis* (Oliv.) Diels	√
13	19.37	Senkyunolide I	Pos	193.1247	193 [M+H]^+^; 147 [M+H-H_2_O-CO]^+^	C_12_H_16_O_2_	*Angelica sinensis* (Oliv.) Diels	19.37
14	22.49	Phthalic anhydride	Pos	149.1343	149 [M+H]^+^	C_8_H_4_O_3_	*Ligusticum chuanxiong* Hort.	√
15	23.45	Cnidilide	Pos	195.1397	195 [M+H]^+^; 149 [M+H-H_2_O-CO]^+^	C_12_H_18_O_2_	Both	√
16	23.61	Ligustilide	Pos	191.1089	191 [M+H]^+^; 149 [M+H-C_3_H_6_]^+^	C_12_H_14_O_2_	Both	√
17	26.14	Neoligustilide	Pos	191.1089	191[M+H]^+^; 149[M+H-C_3_H_6_]^+^	C_12_H_14_O_2_	*Ligusticum chuanxiong* Hort.	√
18	27.08	Diligustilide	Pos	381.2098	381[M+H]^+^	C_24_H_28_O_4_	*Angelica sinensis* (Oliv.) Diels	—

## Data Availability

All experimental data used to support the findings of this study have not been made available because this study is a cooperative enterprise project and has confidentiality agreements to protect raw data. The data used to support this study are available from the corresponding author upon request.
